# Screening and Qualification for Transcatheter Tricuspid Valve Interventions—Preliminary Findings from the CAPTURE Pilot Study

**DOI:** 10.3390/life16040602

**Published:** 2026-04-04

**Authors:** Adam Rdzanek, Adam Piasecki, Ewa Pędzich, Ewa Ostrowska, Paweł Pawłowicz, Ewa Borowiak, Agnieszka Kapłon-Cieślicka, Janusz Kochman, Mariusz Tomaniak, Piotr Scisło, Francesco Maisano

**Affiliations:** 1 1st Chair and Department of Cardiology, Medical University of Warsaw, 02-097 Warsaw, Poland; 2 Department of Cardiac Surgery, San Raffaele University Hospital, 20132 Milan, Italy

**Keywords:** heart failure, transcatheter edge-to-edge repair, transcatheter tricuspid valve interventions, tricuspid regurgitation

## Abstract

**Background:** Transcatheter tricuspid edge-to-edge repair (T-TEER) is the most widely used treatment option for patients with tricuspid regurgitation (TR). In real-world practice, a substantial proportion of referred patients are not eligible for T-TEER or do not achieve an adequate early TR reduction and may therefore require alternative transcatheter tricuspid valve interventions (TTVI)—orthotopic or heterotopic tricuspid valve implantation. The aim of the study was to characterize patients with severe TR referred for transcatheter treatment, and identify patients in whom alternative TTVI strategies may be required. **Methods:** The CAPTURE Study (NCT 06838611) enrolls consecutive patients referred for TR treatment. All patients undergo clinical and echocardiographic assessment to determine eligibility for T-TEER. Candidates for alternative TTVI strategies were defined as patients disqualified from T-TEER due to anatomical ineligibility or those with unsuccessful T-TEER, defined as next-day TTE showing TR more than moderate. This pilot analysis includes patients enrolled from November 2023 to December 2024. **Results:** 147 patients were enrolled, 77 (52.4%) patients were qualified for T-TEER and the procedure was performed in 71 (48.3%) patients, with successful TR reduction in 55 cases (77.5% of treated patients); a subset of 34 patients (23.1%) was identified as potential candidates for alternative TTVI strategies. These patients exhibited more advanced TR (torrential TR 76.5% vs. 18.2%; *p* < 0.001) and right heart failure symptoms (ascites 44.1% vs. 12.7%; *p* < 0.001). Additionally, they had significantly higher bilirubin concentration (1.09 [1.20] mg/dL vs. 0.61 [0.42] mg/dL; *p* = 0.003), lower hemoglobin level (11.8 [1.7] g/dL vs. 12.3 [1.7] g/dL; *p* = 0.017) and platelet count (161.0 [51.0] × 109/L vs. 183.0 [79.0] × 109/L; *p* = 0.015), suggesting an increased bleeding risk. **Conclusions:** In this preliminary single-center real-world cohort, approximately half of the patients with severe TR were eligible for T-TEER, whereas more than 20% emerged as potential candidates for alternative TTVI strategies. This subgroup was characterized by more advanced right-sided remodeling and laboratory features suggestive of hepatic dysfunction and increased bleeding risk, which may have important implications for Heart Team decision-making and procedural planning.

## 1. Introduction

Tricuspid regurgitation (TR) is a common acquired valvular heart disease, primarily affecting the elderly and often leading to disabling symptoms [1]. In recent years, tricuspid transcatheter edge-to-edge repair (T-TEER) has become a widely available treatment option for patients with TR. This approach, characterized by a favorable safety profile, has been proven to reduce the severity of clinical symptoms more effectively than pharmacological therapy [2].

As a result, there has been a significant increase in the number of patients with TR being referred to tertiary care centers for evaluation and consideration for interventional treatment. However, not all referred patients ultimately derive clinical benefit from the T-TEER procedure. During the initial assessment, a substantial proportion of patients are deemed ineligible for T-TEER, mainly due to advanced stage of heart failure or anatomical constraints. Additionally, in some patients undergoing transcatheter valve repair, the procedure fails to achieve the expected reduction in regurgitation severity. Patients who are ineligible for T-TEER due to anatomical limitations or in whom the procedure does not result in sufficient regurgitation reduction may be candidates for alternative transcatheter tricuspid valve interventions (TTVI) such as orthotopic and heterotopic tricuspid valve implantation [3,4].

Up to now, most studies focused on patients undergoing T-TEER and comprehensive data on the overall characteristics of all patients referred to tertiary centers for evaluation and qualification for interventional treatment of TR remains limited. Furthermore, the population of patients who may benefit from alternative TTVI strategies other than T-TEER has not been clearly defined. Consequently, both the demand for these alternative procedures and the identification of clinical phenotypes associated with the need for alternative TTVI strategies require further investigation.

Therefore, in this single-center prospective observational study, we aim to:

(1) provide a characterization of patients with severe or greater TR referred to a tertiary care center for transcatheter treatment
(2) assess the immediate outcomes of treatment in patients selected for T-TEER
(3) characterize the group of patients in whom alternative TTVI strategies may be required

## 2. Material and Methods

### 2.1. Study Design

This single-center prospective observational study (ChAracterization of Patients and Treatment oUtcomes in severe tricuspid regurgitation—CAPTURE; NCT 06838611) enrolls consecutive patients that have been referred to the 1st Chair and Department of Cardiology, Medical University of Warsaw for evaluation and consideration for interventional TR treatment. Eligible patients have at least severe TR confirmed in a transthoracic echocardiography (TTE), are over 18 years old and have signed an informed consent form. Patients who, at baseline, presented with acute coronary syndrome, cardiogenic shock and a known disseminated malignancy are excluded from the study. Prior tricuspid surgery or previous T-TEER/TTVI were not considered exclusion criteria; however, no patients with these characteristics were present in the preliminary cohort. The enrollment started in November 2023 and is still ongoing. Presented data is a preliminary analysis of the patients enrolled until the end of December 2024.

The study design has been previously reported [5]. In brief, all of the patients followed the same decision pathway, described below. After admission, the patients underwent a thorough clinical evaluation and optimization of medical treatment. The severity of TR was confirmed on TTE and categorized according to the five-grade scale. Since only patients with at least severe TR were eligible, all study participants fell into one of the following categories: severe, massive, or torrential. Subsequently, each patient underwent transesophageal echocardiography for the assessment of the tricuspid valve anatomy and T-TEER feasibility [6]. The anatomical assessment included evaluation of key parameters relevant for T-TEER feasibility, including coaptation gap, leaflet morphology and tethering, and potential interaction with right-ventricular leads, with final eligibility determined by Heart Team consensus. Based on this data, during a Heart Team evaluation, patients were either: (1) qualified for T-TEER, (2) disqualified from T-TEER or (3) qualified for another, non-tricuspid-valve-dedicated procedure, that was deemed necessary prior to the treatment of TR. The patients were disqualified from T-TEER based on: (1) lack of heart failure symptoms (asymptomatic TR); (2) clinical futility of the procedure (e.g., poor mobility, advanced frailty, end stage heart failure or other conditions limiting the expected survival); (3) tricuspid valve anatomy not suitable for T-TEER (e.g., large coaptation gap, short or tethered leaflets, severe leaflet degeneration, clear interaction with right-ventricular lead); (4) TEE visualization not sufficient for the T-TEER attempt. Patients who were initially qualified for another cardiovascular intervention prior to TR treatment (e.g., mitral intervention) underwent a secondary clinical and echocardiographic assessment 1–3 months after completion of the prerequisite procedure, to reassess TR severity and eligibility for subsequent transcatheter TR therapy. For this analysis, we defined a prespecified subgroup of patients in whom alternative TTVI strategies may be required during the screening process. This subgroup comprised patients disqualified from T-TEER due to anatomical ineligibility as well as those who underwent T-TEER but did not achieve adequate early TR reduction. For this preliminary analysis, the unsuccessful T-TEER was defined as TR severity greater than moderate in a TTE performed on the day following the procedure. This early echocardiographic assessment reflects routine clinical practice, in which the immediate post-procedural result often determines the need for further therapeutic considerations.

Protocol of the study was accepted by the Ethics Committee of Medical University of Warsaw. All enrolled patients will sign an informed consent form. The study is conducted according to good clinical practice, the Declaration of Helsinki and in compliance with local legal requirements.

### 2.2. Statistical Analysis

Statistical analysis was carried out with IBM SPSS Statistics package (version 29.0; IBM, New York, NY, USA). The Shapiro–Wilk test was performed to assess distribution of the continuous variables. The normally distributed variables are presented as mean and standard deviation (SD) and compared with the *t* test. The non-normally distributed variables are presented as median with interquartile range (IQR) and compared with the Mann–Whitney test. Categorical variables are presented as a number and percentage and compared using the χ^2^ test or the Fisher’s exact test. Statistical significance was established at 2-sided *p*-Value below 0.05. No formal adjustment for multiple comparisons was performed, and therefore the results should be interpreted as exploratory and hypothesis-generating.

## 3. Results

### 3.1. Overall Study Group

From November 2023 to the end of December 2024, a total of 147 patients with severe TR were enrolled in the study. The baseline characteristics of the studied group are summarized in Table 1. Overall, 70 (47.6%) patients were disqualified from T-TEER. The most common causes for disqualification were: asymptomatic TR in 24 (16.3%) patients, clinical futility in 21 (14.3%) patients, anatomical ineligibility in 18 (12.2%) patients, TR grade reduction after initial treatment in 5 (3.4%) patients and lack of adequate TEE visualization for T-TEER attempt in 2 (1.4%) patients.

**Figure 1:**
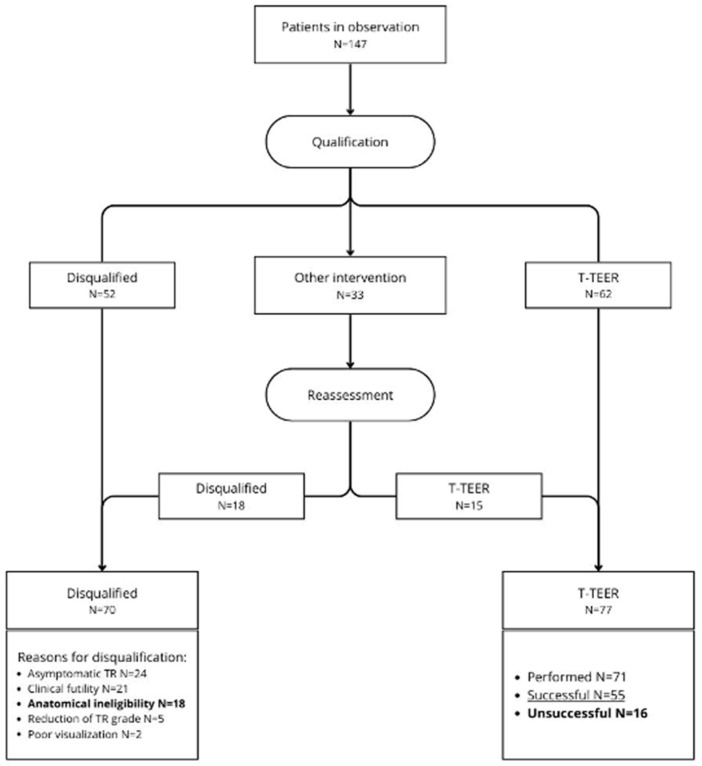
Real-world screening and clinical pathway for patients referred for transcatheter treatment of tricuspid regurgitation, illustrating sequential clinical decision points and management steps. T-TEER—tricuspid transcatheter edge-to-edge repair; TR—tricuspid regurgitation.

**Figure 2:**
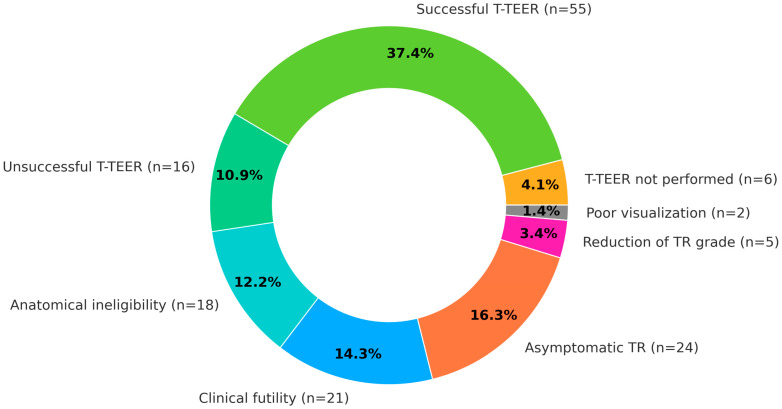
Qualification and immediate treatment outcomes in overall studied population. T-TEER—tricuspid transcatheter edge-to-edge repair.

Out of the 77 (52.4%) patients qualified for T-TEER, 71 (48.3%) underwent the procedure during the observation period, and 6 initially qualified patients did not undergo T-TEER. Successful TR reduction was achieved in 55 (37.4% of the total population and 77.5% of treated with T-TEER) patients. The detailed decision pathway and decision summary are presented in Figure 1 and Figure 2.

### 3.2. Patients in Whom Alternative TTVI Strategies May Be Required

**Figure 3:**
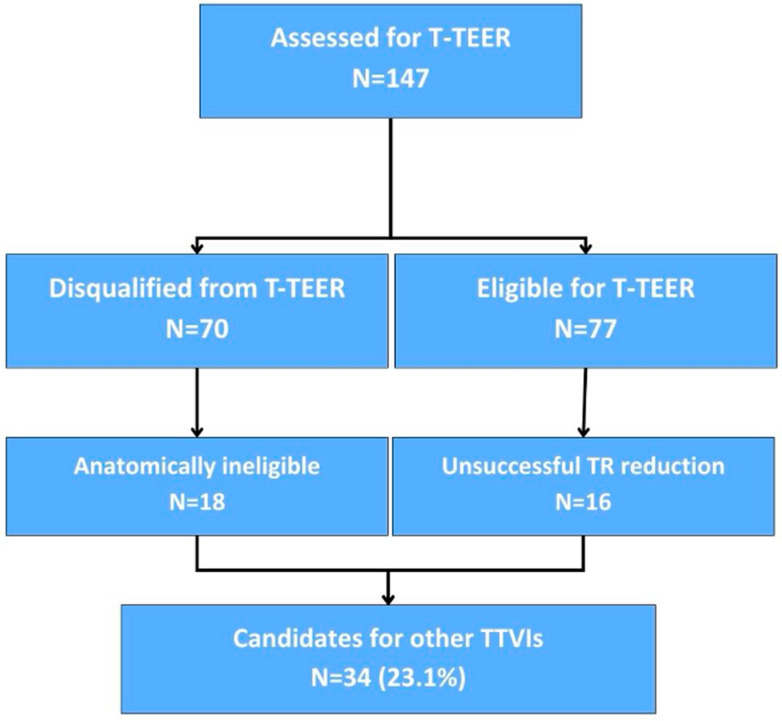
Identification of patients in whom alternative transcatheter tricuspid valve interventions (TTVI) other than tricuspid transcatheter edge-to-edge repair (T-TEER) may be required.

We identified a distinct subgroup of patients in whom alternative TTVI strategies other than T-TEER may be required comprising 18 patients anatomically ineligible for T-TEER and 16 patients with unsuccessful T-TEER attempt, 34 patients in total. The process is presented in Figure 3. Most common anatomical reason for T-TEER ineligibility was a large coaptation gap, that was present in 13 patients. Other reasons included a possible interaction with right-ventricular lead of a cardiac implantable device (four patients) and leaflet anatomy unsuitable for TEER device placement (one patient). A large central coaptation deficit was also one of the main causes for T-TEER failure, identified in 4 out of 16 unsuccessful cases. Moreover, in four cases, despite satisfactory periprocedural TR reduction in TEE, next-day TTE revealed greater than moderate TR grade.

### 3.3. Clinical Characteristics

We compared the candidates for alternative TTVI strategies (*n* = 34) with a group that has undergone a successful T-TEER (*n* = 55). Potential candidates for alternative TTVI strategies were on average younger, with a mean age of 76.5 (7.0) years.

Similarly to the overall study population, most of these patients suffered from severe HF symptoms, with 26 (76.5%) in NYHA functional class III or IV. In comparison, in the successful T-TEER group, there were 37 (67.3%) patients presenting with NYHA III/IV but the difference was not statistically significant. However, in potential candidates for alternative TTVI strategies, significantly more patients presented with ascites—15 (44.1%), compared to 7 (12.7%) in the successful T-TEER group (*p* < 0.001).

The most common comorbidities were atrial fibrillation (AF), chronic kidney disease (CKD) and hypertension. There were no differences in terms of coexisting disease. Furosemide was used more frequently in patients identified as potential candidates for alternative TTVI strategies compared with those successfully treated with T-TEER (79.4% vs. 52.7%, *p* = 0.011), which may reflect a greater burden of congestion and more advanced heart failure in this subgroup.

We found some notable differences in baseline laboratory tests. The potential alternative TTVI strategies candidates had a significantly lower hemoglobin concentration (11.8 [1.7] g/dL vs. 12.3 [1.7] g/dL; *p* = 0.017), lower platelet count (161.0 [51.0] vs. 183.0 [79.0]; *p* = 0.015), higher international normalized ratio (INR; 1.53 [0.88] vs. 1.23 [0.61]; *p* = 0.012) and higher bilirubin concentration (1.09 [1.2] mg/dL vs. 0.61 [0.42] mg/dL; *p* < 0.003). The baseline characteristics of these groups are presented in detail in Table 2.

### 3.4. Echocardiographic Characteristics

At baseline TTE, the candidates for alternative TTVI strategies had a greater severity of TR than those with successful T-TEER attempt. Torrential TR was present in 26 (76.5%) of them, compared to 10 (18.2%) in the successful T-TEER group (*p* < 0.001). Moreover, the dimensions of the right heart chambers were significantly higher as well. Right ventricle diastolic diameter was 4.1 (0.8) cm compared to 3.6 (0.6) cm (*p* < 0.001), right-ventricular inflow tract in apical-four chamber-view was 5.4 (0.9) cm compared to 4.6 (0.6) cm (*p* = 0.005) and right-atrial area was 37.5 (10.1) cm^2^ compared to 34.0 (9.1) cm^2^ (*p* = 0.003). Detailed echocardiographic characterization is presented in Table 2.

## 4. Discussion

This single-center prospective observational study summarizes our initial experience in the identification of TR patients for interventional treatment in real-world clinical practice. It does not aim to define indications or contraindications for specific transcatheter techniques, but to describe screening outcomes in patients referred for transcatheter TR therapies. Importantly, all classifications presented in this study reflect analytical categorization of patients based on real-world clinical decisions and outcomes, rather than predefined treatment algorithms. The main findings of this study are as follows:

First, referral and qualification pattern—patients with severe or greater TR referred to a tertiary care center for transcatheter treatment represent a heterogeneous cohort of elderly individuals with a high burden of heart failure symptoms and multiple comorbidities. Ultimately, about half of these patients is qualified for T-TEER.

Second, immediate outcomes of T-TEER—among patients who underwent T-TEER in our study population, approximately three-quarters experienced an immediate benefit of a significant TR reduction to moderate or less, as assessed by transthoracic echocardiography (TTE) performed on the day following the procedure. Although mid-term and long-term outcomes are essential to assess clinical efficacy, immediate post-procedural TR reduction represents a critical decision point in daily practice, determining whether additional procedures are required.

Third, patients in whom alternative TTVI strategies may be required represent a clinically and echocardiographically distinct phenotype of advanced right-sided heart failure, however this observation should be interpreted cautiously given the limited sample size of this subgroup. Patients that cannot benefit from T-TEER constitute almost one quarter of patients referred to tertiary care center for evaluation and consideration for interventional treatment. Since T-TEER is usually considered as a first-line percutaneous treatment, these patients are candidates for alternative TTVI strategies. Compared to patients who derive immediate benefit from T-TEER, this group is clinically characterized by a higher prevalence of right heart failure symptoms and a distinct laboratory profile including lower hemoglobin levels, lower platelet count, higher INR, and higher bilirubin concentration. These findings may reflect several mechanisms, including hepatic congestion related to advanced right-sided heart failure or the effects of chronic anticoagulation therapy. Importantly, since bleeding outcomes were not assessed in this cohort, these laboratory abnormalities should be interpreted cautiously and cannot be considered direct predictors of bleeding risk. In echocardiography, this population exhibits a greater TR severity which is accompanied by greater right heart chamber dilation. Importantly, these patients emerged naturally from the real-world screening and treatment process and these findings should not be interpreted as contraindications to transcatheter intervention, but rather as markers of advanced disease that may influence the choice and timing of TTVI strategies. It should be emphasized that the grouping of patients anatomically ineligible for T-TEER and those with unsuccessful T-TEER reflects a pragmatic approach used to identify patients who may require alternative TTVI strategies at an early stage of the treatment pathway. It should also be acknowledged that group of candidates for alternative TTVI strategies represents a heterogeneous population, and although certain anatomical determinants, such as a large coaptation gap, may partially overlap between patients disqualified from T-TEER for anatomical reasons and those with unsuccessful T-TEER, this heterogeneity may introduce bias in comparisons and should be considered when interpreting the results.

In recent years, we have witnessed the rapid advancement of transcatheter techniques for the treatment of TR. Due to its favorable safety profile, simplicity, and prior experience with the mitral valve, transcatheter edge-to-edge repair has become the most widely utilized interventional modality in the TR population. Despite anatomical and technical obstacles, there has also been extensive research on the development of transcatheter tricuspid valve prostheses. The EVOQUE system (Edwards Lifesciences, Irvine, CA, USA), designed for orthotopic tricuspid valve implantation, is the first device of its kind to be introduced for commercial use in Europe and the United States. In a recent randomized study, it was shown to be effective in symptom reduction and the quality-of-life improvement when compared to medical therapy alone [7]. Different technique, orthotopic caval valve implantation, may also play a role in the treatment of patients with TR. The TricValve system (P & F Products & Features GmbH, Munich, Germany), designed for this procedure, has been evaluated in the recent TricBicaval registry, demonstrating improvements in patients’ functional status [8]. Systems for orthotopic and heterotopic valve implantation may be used both in patients who are not eligible for T-TEER and in those who have undergone an unsuccessful transcatheter tricuspid valve repair.

To date, there are no definitive guidelines for patient selection among different transcatheter techniques. Therefore, the qualification process primarily relies on an individualized assessment of each patient’s anatomy and risk-to-benefit ratio, with T-TEER being the preferred interventional modality due to its favorable safety profile. Furthermore, qualification for interventional treatment may vary significantly between centers due to differences in operator experience and the availability of specific techniques.

Moreover, until now, only a few published studies focused on the selection of patients for invasive TR treatment. In a retrospective analysis involving 547 patients from three centers, 196 (35.8%) patients were qualified for T-TEER, while a total of 136 (24.9%) patients were referred to other transcatheter therapeutic modalities, mainly direct annuloplasty and, in minority of cases, transcatheter valve implantation [9]. In the latter group, larger right heart dimensions with concomitant larger TR severity were observed, indicating more advanced stage of disease in candidates for interventions other than T-TEER.

In another retrospective study involving patients evaluated for tricuspid interventions, anatomical feasibility for T-TEER and transcatheter tricuspid valve implantation were analyzed [10]. Among 491 patients assessed for T-TEER, 157 (32.0%) were found to have unfavorable anatomy for percutaneous valve repair attempt. Also, in this case, unfavorable T-TEER candidates were characterized by more severe TR and more pronounced dilatation of the right atrium and right ventricle. It should also be emphasized that in the group of patients ineligible for T-TEER who underwent evaluation by computed tomography, 69% were also not eligible for valve implantation.

A recent analysis of a real-life population showed that, out of 178 patients with TR referred to the tertiary center, only 19 (10.7%) were eligible for the enrolment to the clinical trials on transcatheter TR treatment (T-TEER or alternative TTVI strategies) and an additional 36 (20.2%) underwent out-of-trial T-TEER [11].

Retrospective studies on patient selection for transcatheter TR treatment published so far have differed in methodology, focused on the eligibility for different interventional techniques (annuloplasty, tricuspid valve implantation) or have analyzed study populations based on an intention-to-treat basis. Unlike prior retrospective analyses focused on anatomical feasibility, the present study prospectively captures the full clinical pathway, including patients with unsuccessful T-TEER, reflecting real-world decision-making. In daily clinical practice, alternative TTVI strategies are considered not only in patients disqualified from T-TEER due to anatomical reasons but also in those without a significant TR reduction after T-TEER. Our findings in this group confirm previous observations showing larger right heart diameters and more severe TR grade when compared to the successful T-TEER patients.

The higher prevalence of laboratory markers associated with increased bleeding risk in patients requiring alternative TTVI strategies represents an important clinical observation from the presented analysis. In our opinion, this observation may have clinical implications. It is also consistent with the excessive bleeding rates reported in EVOQUE and TricValve series and highlights that comprehensive pre-procedural bleeding risk stratification should represent an important element of Heart Team discussions when considering TTVI strategies. As the CAPTURE registry is an ongoing prospective study, future analyses will include echocardiographic assessment and predefined clinical endpoints, such as functional status, heart failure hospitalizations, mortality, and quality of life, assessed at 1- and 12-month follow-up, with planned extension for long-term outcome evaluation.

## 5. Conclusions

In real-world practice, approximately half of patients with severe tricuspid regurgitation referred for transcateter treatment are eligible for T-TEER, whereas more than 20% emerge as potential candidates for alternative TTVI strategies. This subset is characterized by more advanced disease and a laboratory profile suggestive of increased bleeding risk, which should be carefully considered during Heart Team decision-making.

### Study Limitations

Several limitations of this study should be emphasized. First, although prospective in design, this registry is a single-center analysis based on patient referrals from primary care centers and outpatient clinics, making it susceptible to selection bias and potentially not fully representative of the broader TR patient population. Second, the number of T-TEER procedures performed during the first year of observation was limited, and not all qualified patients underwent treatment. This may have influenced the proportion of patients benefiting from the procedure in terms of TR reduction, thereby altering the number of potential candidates for alternative TTVI strategies. Third, the significant TR reduction observed in our T-TEER group (76.6%) is consistent with findings from large-scale registries [12]. However, we used a different time point to assess post-procedural TR, reporting immediate results based on TTE performed the day after the procedure, rather than at 30 days. Another limitation is the relatively small number of patients identified as potential candidates for alternative TTVI strategies, which limits the statistical power of comparisons and precludes definitive conclusions. Finally, in this preliminary report we focused on clinical and basic echocardiographic data obtained during the initial screening process, without providing a detailed analysis of tricuspid valve anatomy (such as coaptation gap, leaflet morphology, or annular dimensions). A comprehensive anatomical characterization will be addressed in subsequent analyses of the registry. Therefore, the present analysis should be interpreted as hypothesis-generating and descriptive, providing a framework for future multicenter validation within the CAPTURE registry.

## Figures and Tables

**Table 1:** Baseline characteristics of overall studied population.

Parameters	N = 147
Clinical characteristics	
Age, years	78.0 (10.0)
Female, n (%)	90 (61.2)
NYHA III/IV	101 (68.7)
Peripheral edema	104 (70.7)
Ascites	36 (24.5)
Previous HF hospitalization	129 (87.8)
At least 1 HHF in past 12 months	91 (61.9)
Concomitant disease	
AF	105 (71.4)
CAD	63 (42.9)
PAD	18 (12.2)
Hypertension	110 (74.8)
DM	38 (25.9)
DM insulin	5 (3.4)
CKD	108 (73.5)
COPD/asthma	24 (16.3)
Past medical history	
Previous MI	35 (23.8)
Previous stroke/TIA	16 (10.9)
PCI	32 (21.8)
CABG	14 (9.5)
AVR	10 (6.8)
MVR	12 (8.2)
TVP	1 (0.7)
PM	53 (36.1)
ICD	11 (7.5)
CRT	8 (5.4)
Baseline pharmacotherapy	
Furosemide *	89 (60.5)
Torasemide *	114 (77.6)
Hydrochlorothiazide	27 (18.4)
Spironolactone	31 (21.1)
Eplerenone	75 (51.0)
BB	134 (91.2)
ACE-i	76 (51.7)
ARB	11 (7.5)
ARNI	6 (4.1)
CCB	11 (7.5)
ASA	9 (6.1)
P2Y12i	9 (6.1)
VKA	23 (15.6)
NOAC	104 (70.7)
Statin	85 (57.8)
SGLT-2 inhibitors	88 (59.9)

* In selected patients with advanced heart failure, concomitant use of two loop diuretics was required to achieve adequate symptom control. Abbreviations: ACE-i—angiotensin-converting enzyme inhibitor; AF—atrial fibrillation; ARB—angiotensin receptor blocker; ARNI—angiotensin receptor–neprilysin inhibitor; ASA—acetylsalicylic acid; AVR—aortic valve replacement; BB—beta-blocker; CABG—coronary artery bypass grafting; CAD—coronary artery disease; CCB—calcium channel blocker; CKD—chronic kidney disease; COPD—chronic obstructive pulmonary disease; CRT—cardiac resynchronization therapy; DM—diabetes mellitus; HF—heart failure; HHF—hospitalization for heart failure; ICD—implantable cardioverter-defibrillator; MI—myocardial infarction; MVR—mitral valve repair/replacement; NOAC—non-vitamin K antagonist oral anticoagulant; NYHA—New York Heart Association; PAD—peripheral artery disease; PCI—percutaneous coronary intervention; PM—pacemaker; P2Y12i—P2Y12 inhibitor; SGLT-2—sodium–glucose cotransporter 2; TIA—transient ischemic attack; TVP—tricuspid valve procedure; VKA—vitamin K antagonist.

**Table 2:** Characteristics of the patients divided into two groups (1) patients successfully treated with T-TEER, i.e., with post-procedural TR grade moderate or less and (2) patients disqualified from T-TEER due to anatomical ineligibility combined with patients with unsuccessful T-TEER.

Parameters	Overall N = 89	Group 1 Successful T-TEER N = 55	Group 2 Potential Alternative TTVI Strategies Candidates N = 34	*p*-Value
Clinical characteristics				
Age, years	78.0 (9.0)	78.7 (6.6)	76.5 (7.0)	0.045
Female, n (%)	56 (62.9)	37 (67.3)	19 (55.9)	0.280
NYHA III/IV	63 (70.8)	37 (67.3)	26 (76.5)	0.354
Peripheral edema	63 (70.8)	37 (67.3)	26 (76.5)	0.354
Ascites	22 (24.7)	7 (12.7)	15 (44.1)	<0.001
Previous HF hospitalization	79 (88.8)	47 (85.5)	32 (94.1)	0.209
At least 1 HHF in past 12 months	55 (61.8)	34 (61.8)	21 (61.8)	0.996
Concomitant disease				
AF	82 (92.1)	51 (92.7)	31 (91.2)	0.999
CAD	35 (39.3)	22 (40.0)	13 (38.2)	0.868
PAD	10 (11.2)	8 (14.5)	2 (5.9)	0.209
Hypertension	64 (71.9)	41 (74.5)	23 (67.6)	0.482
DM	23 (25.8)	17 (30.9)	6 (17.6)	0.165
CKD	67 (75.3)	41 (74.5)	26 (76.5)	0.838
COPD/asthma	12 (13.5)	8 (14.5)	4 (11.8)	0.999
Past medical history				
Previous MI	21 (23.6)	15 (27.3)	6 (17.6)	0.299
Previous stroke/TIA	12 (13.5)	10 (18.2)	2 (5.9)	0.121
PCI	16 (18.0)	12 (21.8)	4 (11.8)	0.230
CABG	7 (7.9)	4 (7.3)	3 (8.8)	0.999
AVR	6 (6.7)	4(7.3)	2 (5.9)	0.999
MVR	9 (10.1)	4(7.3)	5 (14.7)	0.258
TVP	1 (1.1)	0 (0.0)	1 (2.9)	0.382
PM	34 (38.2)	21 (38.2)	13 (38.2)	0.996
ICD	8 (9.0)	6 (10.9)	2(5.9)	0.705
CRT	4 (4.5)	4 (7.3)	0 (0.0)	0.293
Baseline pharmacotherapy				
Furosemide *	56 (62.9)	29 (52.7)	27 (79.4)	0.011
Torasemide *	67 (75.3)	43 (78.2)	24 (70.6)	0.420
Hydrochlorothiazide	14 (15.7)	7 (12.7)	7 (20.6)	0.322
Spironolactone	20 (22.5)	13 (23.6)	7 (20.6)	0.738
Eplerenone	49 (55.1)	27 (49.1)	22 (64.7)	0.150
BB	82 (92.1)	52 (94.5)	30 (88.2)	0.421
ACE-i	44 (49.4)	26 (47.3)	18 (52.9)	0.603
ARB	7 (7.9)	6 (10.9)	1 (2.9)	0.244
ARNI	2 (2.2)	2 (3.6)	0 (0.0)	0.522
CCB	7 (7.9)	3 (5.5)	4 (11.8)	0.421
ASA	4 (4.5)	4 (7.3)	0 (0.0)	0.293
P2Y12 inhibitors	3 (3.4)	3 (5.5)	0 (0.0)	0.284
VKA	17 (19.1)	9 (16.4)	8 (23.5)	0.403
NOAC	64 (71.9)	39 (70.9)	25 (73.5)	0.789
Fractioned heparin	1 (1.1)	1 (1.8)	0 (0.0)	0.999
Statin	51 (57.3)	31 (56.4)	20 (58.8)	0.820
SGLT-2 inhibitors	57 (64.0)	35 (63.6)	22 (64.7)	0.919
Baseline laboratory tests				
Hemoglobin (g/dL)	12.4 (1.8)	12.3 (1.7)	11.8 (1.7)	0.017
Platelet count (×10^3^/L)	177.0 (60.0)	183.0 (79.0)	161.0 (51.0)	0.015
CRP (mg/L)	2.5 (4.5)	2.1 (4.3)	3.7 (6.1)	0.092
Urea (mg/dL)	64.0 (34.0)	61.0 (40.0)	65.0 (32.0)	0.584
Creatinine (mg/dL)	1.36 (0.52)	1.42 (0.42)	1.29 (0.46)	0.450
eGFR (mL/min/1.73 m^2^)	42.0 (24.0)	40.0 (23.0)	46.8 (17.1)	0.321
AST (U/L)	31.5 (10.0)	31.5 (10.0)	31.5 (10.0)	0.600
ALT (U/L)	23.0 (11.0)	22.5 (11.0)	23.0 (10.0)	0.901
INR	1.31 (0.81)	1.23 (0.61)	1.53 (0.88)	0.012
Bilirubin (mg/dL)	0.72 (0.54)	0.61 (0.42)	1.09 (1.20)	0.003
NT-proBNP (pg/mL)	1542.0 (1631.0)	1542.0 (1602.0)	1500.5 (1559.0)	0.775
Sodium (mmol/L)	139.1 (3.8)	139.6 (4.3)	138.5 (3.6)	0.039
Baseline echocardiography				
MR				
- no/trace	15 (16.8)	4 (7.3)	5 (14.7)	0.247
- mild	39 (43.8)	21 (38.2)	18 (52.9)	
- moderate	27 (30.3)	21 (38.2)	6 (17.6)	
- moderate-severe	2 (2.2)	1 (1.8)	1 (2.9)	
- severe	6 (6.7)	3 (5.5)	3 (8.8)	
TR				
- severe	21 (23.6)	20 (36.4)	1 (2.9)	<0.001
- massive	32 (36.0)	25 (45.5)	7 (20.6)	
- torrential	36 (40.4)	10 (18.2)	26 (76.5)	
LVDd, cm	4.9 (1.1)	4.8 (1.2)	5.1 (0.7)	0.623
IVSd, cm	1.0 (0.3)	1.0 (0.2)	1.0 (0.3)	0.773
PWTd, cm	1.0 (0.2)	1.0 (0.2)	1.0 (0.2)	0.834
RVDd, cm	3.7 (0.2)	3.6 (0.6)	4.1 (0.8)	<0.001
LA, cm	5.2 (0.9)	5.2 (1.0)	5.3 (0.9)	0.280
LVEF, %	57.0 (16.0)	57.0 (18.0)	55.0 (8.0)	0.389
RAA, cm^2^	37.4 (10.2)	34.0 (9.1)	37.5 (10.1)	0.003
RVIT, cm	5.0 (0.8)	4.6 (0.6)	5.4 (0.9)	0.001
TAPSE, mm	17.1 (4.6)	17.4 (4.8)	16.6 (4.4)	0.465
TR ERO, cm^2^	0.73 (0.33)	0.62 (0.23)	0.93 (0.50)	<0.001
TR Vol, mL	64.0 (30.0)	57.0 (23.0)	80.0 (29.0)	<0.001

* In selected patients with advanced heart failure, concomitant use of two loop diuretics was required to achieve adequate symptom control. Abbreviations: AF—atrial fibrillation; ARB—angiotensin receptor blocker; ARNI—angiotensin receptor–neprilysin inhibitor; ASA—acetylsalicylic acid; AVR—aortic valve replacement; BB—beta-blocker; CABG—coronary artery bypass grafting; CAD—coronary artery disease; CCB—calcium channel blocker; CKD—chronic kidney disease; COPD—chronic obstructive pulmonary disease; CRT—cardiac resynchronization therapy; DM—diabetes mellitus; ERO—effective regurgitant orifice; HF—heart failure; HHF—hospitalization for heart failure; ICD—implantable cardioverter-defibrillator; INR—international normalized ratio; LA—left atrium; LVEF—left-ventricular ejection fraction; MI—myocardial infarction; MR—mitral regurgitation; MVR—mitral valve repair/replacement; NOAC—non-vitamin K antagonist oral anticoagulant; NT-proBNP—N-terminal pro–B-type natriuretic peptide; NYHA—New York Heart Association; PAD—peripheral artery disease; PCI—percutaneous coronary intervention; PM—pacemaker; P2Y12 inhibitors—P2Y12 receptor inhibitors; RAA—right-atrial area; RVDd—right-ventricular diastolic diameter; RVIT—right-ventricular inflow tract; TAPSE—tricuspid annular plane systolic excursion; TIA—transient ischemic attack; TR—tricuspid regurgitation; T-TEER—tricuspid transcatheter edge-to-edge repair; TTVI—transcatheter tricuspid valve intervention; VKA—vitamin K antagonist.

## Data Availability

The data supporting the findings of this study are available from the corresponding author upon reasonable request.

## Abbreviations

6MWD—6 min walk distance; ACE-I—angiotensin-converting enzyme inhibitor; AF—atrial fibrillation; ALT—alanine transaminase; AST—aspartate transaminase; ARB—angiotensin receptor blocker; ASA—acetylsalicylic acid; AVR—aortic valve replacement; CABG—coronary artery bypass grafting; CAD—coronary artery disease; CCB—calcium channel blocker; CKD—chronic kidney disease; COPD—chronic obstructive pulmonary disease; CRP—C-reactive protein; DM—diabetes mellitus; eGFR—estimated glomerular filtration rate; ERO—effective regurgitant orifice; HHF—hospitalization for heart failure; INR—international normalized ratio; KCCQ OS—Kansas City Cardiomyopathy Questionnaire Overall Summary Score; LAA—left atrial area; LVEF—left-ventricular ejection fraction; MI—myocardial infarction; MR—mitral regurgitation; MVR—mitral valve repair/replacement; NOAC—non-vitamin K antagonist oral anticoagulant; NT-proBNP—N-terminal pro-B-type natriuretic peptide; NYHA—New York Heart Association; PAD—peripheral artery disease; PCI—percutaneous coronary intervention; RAA—right-atrial area; RVIT A4C—right-ventricular inflow tract in apical 4-chamber view; RVOT—right-ventricular outflow tract; SLDA—single leaflet device attachment; SPAP—systolic pulmonary artery pressure; TAPSE—tricuspid annular plane systolic excursion; TAVI—transcatheter aortic valve implantation; TIA—transient ischemic attack; TEE—transesophageal echocardiography; TR—tricuspid regurgitation; TTE—transthoracic echocardiography; T-TEER—tricuspid transcatheter edge-to-edge repair; TTVI—transcatheter tricuspid valve intervention; TVG—tricuspid valve gradient; TVR—tricuspid valve repair/replacement; VKA—vitamin K antagonist.
